# Role of BRCA Mutations in Cancer Treatment with Poly(ADP-ribose) Polymerase (PARP) Inhibitors

**DOI:** 10.3390/cancers10120487

**Published:** 2018-12-04

**Authors:** Isabella Faraoni, Grazia Graziani

**Affiliations:** Department of Systems Medicine, University of Rome Tor Vergata, 00173 Rom, Italy

**Keywords:** PARP inhibitors, ovarian cancer, breast cancer, DNA repair, homologous recombination, non-homologous end joining, olaparib, rucaparib, niraparib, talazoparib

## Abstract

Inhibition of poly(ADP-ribose) polymerase (PARP) activity induces synthetic lethality in mutated BRCA1/2 cancers by selectively targeting tumor cells that fail to repair DNA double strand breaks (DSBs). Clinical studies have confirmed the validity of the synthetic lethality approach and four different PARP inhibitors (PARPi; olaparib, rucaparib, niraparib and talazoparib) have been approved as monotherapies for BRCA-mutated or platinum-sensitive recurrent ovarian cancer and/or for BRCA-mutated HER2-negative metastatic breast cancer. PARPi therapeutic efficacy is higher against tumors harboring deleterious germline or somatic BRCA mutations than in BRCA wild-type tumors. BRCA mutations or intrinsic tumor sensitivity to platinum compounds are both regarded as indicators of deficiency in DSB repair by homologous recombination as well as of favorable response to PARPi. However, not all BRCA-mutated or platinum-responsive patients obtain clinical benefit from these agents. Conversely, a certain percentage of patients with wild-type BRCA or platinum-resistant tumors can still get benefit from PARPi. Thus, additional reliable markers need to be validated in clinical trials to select patients potentially eligible for PARPi-based therapies, in the absence of deleterious BRCA mutations or platinum sensitivity. In this review, we summarize the mechanisms of action of PARPi and the clinical evidence supporting their use as anticancer drugs as well as the additional synthetic lethal partners that might confer sensitivity to PARPi in patients with wild-type BRCA tumors.

## 1. Introduction

Different genotoxic agents of physical or chemical origin can alter the natural DNA structure. These include oxidizing agents or ultraviolet radiations, which modify the chemical structure of the DNA nitrogen bases, ionizing radiations, which cause DNA strand breaks, and several anticancer drugs (e.g., crosslinking or reactive oxygen species-generating agents). If the damage is not repaired, DNA mutations and loss of genetic information may occur.

Since genotoxic molecules can also be produced by the normal cellular metabolism, the frequency of DNA alterations in chemical structure is very high. Every day, it has been estimated that each cell experiences on average approximately 10^5^ spontaneous DNA lesions (e.g., oxidized bases, depurination) [1]. Nevertheless, evolution has provided cells with highly efficient DNA damage repair systems capable of removing injuries and correcting subsequent sequence errors. Congenital defects in DNA repair mechanisms may result in accumulation of DNA mutations and increased risk of cancer, as evidenced by the inherited disorders Xeroderma pigmentosum and Ataxia telangiectasia.

The importance of genes implicated in DNA damage response or repair pathways in tumor development was highlighted in the 1990s by two research groups that found an increased susceptibility to inherited breast cancer (BC) and ovarian cancer (OC) in patients harboring germline mutations of BRCA1 and BRCA2 genes [2,3]. Although different in protein sequence and structure, BRCA1 and BRCA2 act in a common pathway of genome protection. Moreover, mutations in BRCA1 or BRCA2 are mutually exclusive, thus they are often referred to as BRCA mutations without distinction between them.

The aim of this review is to summarize the mechanisms of action and current clinical evidence supporting the antitumor activity of poly (ADP-ribose) polymerase (PARP) inhibitors in ovarian and breast cancers with defects in the repair of DNA damage, related or unrelated to BRCA mutations.

## 2. BRCA Genes and Cancer Susceptibility

The BRCA1 gene is located on the long arm of chromosome 17, consisting of 24 exons. A large number of deletions, insertions or duplications have been reported in its sequence, probably due to the high density of Alu elements in the introns (around 41%). Missense, nonsense and silent mutations, splice-site mutations and mutations in untranslated regions can also be found [4]. The BRCA2 gene is located on the long arm of chromosome 13 and is larger than BRCA1, consisting of 27 exons. To date, about 2000 different mutations have been identified in both genes, although not all of them are risk-associated. BRCA genetic alterations known to increase individual cancer susceptibility are referred to as “deleterious” mutations and, in the majority of cases, they introduce premature stop codons leading to truncated and nonfunctional proteins [4,5]. Thus, mutations may disturb BRCA1 and BRCA2 participation in DNA repair or interaction with repair proteins.

Given the complexity of these genes, it is difficult to make an accurate estimate of the increased risk of BC or OC development in BRCA1 or BRCA2 mutation carriers. A recent prospective study on 9856 BRCA1 or BRCA2 mutation carriers reported a cumulative risk of BC and OC to age 80 years of 72% and 44%, respectively, for germline BRCA1 mutations and, 69% and 17% in case of germline BRCA2 mutations [6] These data are consistent with findings from retrospective family-based studies [7]. Conversely, in the general population the cumulative BC and OC risk is 12% and 1.3%, respectively [8]. Moreover, cancer risk varies by mutation location within the BRCA1 or BRCA2 genes and family history [6]. Deleterious BRCA1 or BRCA2 mutations may also moderately increase the risk of breast and prostate cancer in men [9], and pancreatic cancer [10,11] or colorectal cancer [12] in both sexes.

## 3. Role of PARP Enzymes and BRCA Proteins in DNA Repair

In order to maintain genomic integrity, cells are endowed with mechanisms that detect and repair endogenously or exogenously-induced DNA lesions through a coordinated action defined as DNA damage response. Numerous “sensors” of DNA damage are present in the nucleus, which recruit downstream effectors at the sites of interest [13]. Among them, poly(ADP-ribose) polymerase-1 (PARP1) is an abundant nuclear protein activated by DNA breaks capable of synthesizing poly(ADP-ribose) (PAR) chains that serve as a signal and platform for the recruitment of several DNA repair proteins. PARP1 belongs to the superfamily of ADP-ribosyl transferases comprising 17 enzymes that transfer PAR or mono-ADP-ribose to themselves and/or other target proteins. Besides PARP1, also PARP2 and PARP3 possess DNA-dependent (ADP-ribose) transferase activity. While PARP1 and PARP2 catalyze the synthesis of long PAR chains, PARP3 is a mono-ADP transferase.

Poly-ADP-ribosylation (PARylation) consists in the covalent binding of negatively charged PAR on glutamate, aspartate or lysine residues of target proteins [14]. During this reaction, PARPs use oxidized nicotinamide adenine dinucleotide (NAD+) as a substrate, with the release of nicotinamide and a proton, and cell consumes ATP in an effort to restore NAD+ levels. PARylation can produce different effects: it destabilizes or stabilizes protein-DNA interactions, regulates protein-protein interactions and functions, promotes the activity of target proteins but also induces protein degradation by proteasome [15]. Through PARylation, PARP proteins control multiple cellular functions, such as DNA duplication and transcription, and are of great importance in DNA damage response and cell death [16].

The degradation of PAR is also a highly regulated cellular mechanism that is mediated by a class of enzymes known as poly(ADP-ribose) glycohydrolases (PARG), which hydrolyze the glycosidic bonds between ADP-ribose units; the ADP-ribose unit bound to the acceptor protein is instead removed by the ADP-ribosyl protein lyase [17].

**Figure 1:**
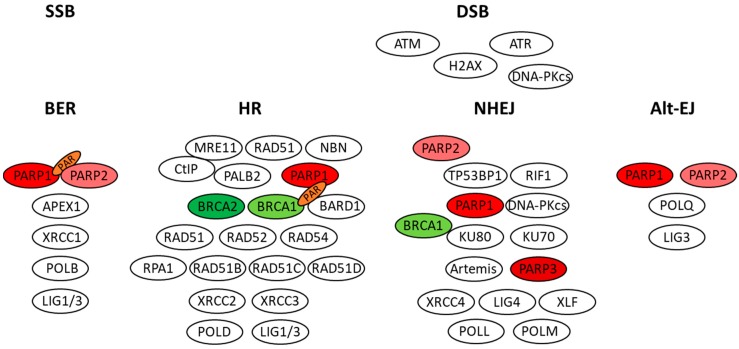
Role of PARPs in BER, HR, NHEJ and Alt-EJ.

PARP1 is responsible for 85–90% of the total cellular PARylation activity that can increases up to 500-fold in response to DNA damage. About 50–200 PAR residues may be attached to PARP1 itself (auto-ribosylation) as well as to other nuclear (acceptor) proteins. PARP1 is able to recognize single strand breaks (SSB) and is involved in the base excision repair (BER) [18]. The damaged or incorrect DNA base is recognized by specific glycosylases that generate an abasic site, which induces the recruitment of the endonuclease APEX1. In turn, APEX1 by removing the nucleotide skeleton generates a single-stranded nick that acts as a recruitment site for PARP1. After recognition of DNA breaks, PARP1 undergoes rapid auto-ribosylation and forms PAR on histone and non-histone proteins by a transesterification reaction. Through PARylation PARP1 also mediates the recruitment to the damaged site of PARP2. Thereafter, PARP1 and PARP2 further increase the signal required for the accumulation of proteins that process and ultimately repair the DNA lesion [19,20,21]. The negatively charged PAR forms a sort of matrix that is able to maintain the DNA conformation in an open state. This allows the access of various factors involved in the repair process [22,23]: XRCC1 that stabilizes DNA breaks, polymerase POLB that fills the nick with the correct base, and ligase LIG1/3 that seals the junctions [24] (Figure 1).

PARP1 can also regulate the function of proteins involved in the repair of DNA double strand breaks (DSB) that is mediated by the homologous recombination (HR) and by the classical non-homologous end joining pathway (NHEJ) (Figure 1).

Following the recognition of the DSB, a complex cascade of reactions is activated in order to recruit the proteins involved in DNA repair. The protein kinases ATM, ATR and DNA-PKcs, phosphorylate different proteins, including histone H2AX on serine 139 (γH2AX) [25]. The formation of γH2AX is a rapid and sensitive response to the presence of DSB as it alters the chromatin structure. Indeed, the appearance of γH2AX cell positivity is frequently used as a marker of DSB.

The ATM kinase and the MRE11-RAD50-NBN (MRN) complex mediate the initial cellular response by HR. The MRN complex has the role of signaling, stabilizing and acting as a scaffold for other proteins involved in DSB repair [26]. Due to its exonuclease activity, MRE11 erodes the 5′ end near the break and the endonuclease CtIP further stimulates resection in the 5′ to 3′ direction to generate 3′ single strand DNA (ssDNA) overhangs. The ssDNA is then coated by RPA1 that protects the DSB ends from further degradation and signals the presence of unrepaired DNA damage. Thereafter, RAD51 and RAD52 bind to the protruding 3′ end, and RAD51 mediates the recognition of the template on the homologous chromosome. Once the homologous DNA has been identified, the damaged strand invades the double strand of intact DNA, ensuring a template-directed DNA synthesis and high-fidelity repair. For this reason, the HR system is an error-free repair pathway. Finally, a DNA polymerase extends the 3′ end and a ligase joins the ends by reforming the double strand.

Both BRCA1 and BRCA2 interact with various proteins involved in the HR repair pathway and appear indispensable for this process, acting at different stages in DSB repair [27]. In particular, BRCA1 can bind DSBs, interact with the MRN complex and contribute to the DSBs processing. Moreover, BRCA1 interacts with BRCA2 via the bridging protein PALB2 during the recruitment of RAD51 to DSBs. The BRCA2 protein binds RAD51 and guides it to the site of DNA damage. In regard to PARP1 involvement in HR, this enzyme has an active role at stalled replication forks, mediating replication restart and favoring the recruitment of MRN components (i.e., MRE11 and NBN) [28]. Furthermore, through PARylation PARP1 modulates the DNA binding activity of BRCA1. This process requires initially PAR recognition at DNA lesions by BARD1 that mediates the rapid recruitment of BRCA1 and, subsequently, the binding of BRCA1-BARD1 complex to γH2AX at the DNA break [29,30].

When a second copy of the DNA is not available (i.e., in quiescent cells) or if the HR system is not functioning because of HR gene mutations, the NHEJ system comes into play. In the presence of DSB, ATM phosphorylates TP53BP1 that recruits RIF1 and blocks CtIP-dependent DNA end resection thus activating the NHEJ pathway. The NHEJ repair simply joins the ends of the DSB, without any template; therefore, it is an error-prone repair mechanism. During the repair process, the KU70-KU80 heterodimer binds to DNA-PKcs and Artemis nuclease, and the complex protects the DNA open ends favoring the recruitment of the XRCC4-LIG4-XLF complex [31,32]. BRCA1 physically interacts with KU80 and the ATM-mediated phosphorylation of BRCA1 seems to be important for the precise end-joining activity by NHEJ [33,34]. Moreover, BRCA1 through interaction with CtIP favors the dissociation of TP53BP1-RIF1 and directs the repair pathway from NHEJ to HR [35]. In regard to PARPs involvement in NHEJ, PARP1 inhibits NHEJ repair by preventing the binding of the KU proteins to free DNA ends and PARP2 favors TP53BP1 accumulation onto broken DNA, facilitating the CtIP-dependent DNA end-resection and HR repair [36,37]. Conversely, PARP3 promotes DSB repair by NHEJ by limiting DNA end resection [38].

In the absence of NHEJ components, an alternative NHEJ (Alt-EJ) contributes to resolve DSBs. The Alt-EJ requires the activity of POLQ polymerase and of proteins involved in other DNA repair systems (BER, MRN complex and inter-strand cross-link repair), including PARP1 [39] (Figure 1).

## 4. PARP Inhibitors Enter the Clinic

PARP inhibitors (PARPi) have been developed to hamper DNA repair by blocking PARP enzyme activity and PARylation reactions. Their mechanism of action requires competition with NAD+ for the interaction with the PARP catalytic domain. The close link between BRCA and PARP1 was highlighted in 2005 when two research groups independently discovered that PARP inhibition induces synthetic lethality in mutated BRCA1 or BRCA2 cancers [40,41].

In the presence of PARPi, the endogenously generated SSB are no longer repaired by BER and are converted into DSB during cell duplication. Cells defective in BRCA function cannot repair the damage and die, whereas normal tissues that retain a single wild-type copy of the relevant gene may still repair DSB by means of a functional HR. Indeed, in cancer patients harboring germline BRCA mutations tumor cells show a biallelic defect and defective HR, while normal tissues remain BRCA proficient.

Recently, other mechanisms involved in PARPi anticancer activity have been described [42]. They include PARP1 trapping on damaged DNA due to the formation of PARP-DNA complexes that prevent DNA replication and transcription. This mechanism is independent on catalytic inhibition and has been attributed to an allosteric conformational change in PARP1 and PARP2 that results in the stabilization of their interaction with DNA [43]. Moreover, PARG inhibition reduces the amount of trapped PARP1 and the accumulation of toxic PARP1-DNA complexes [44].

**Figure 2:**
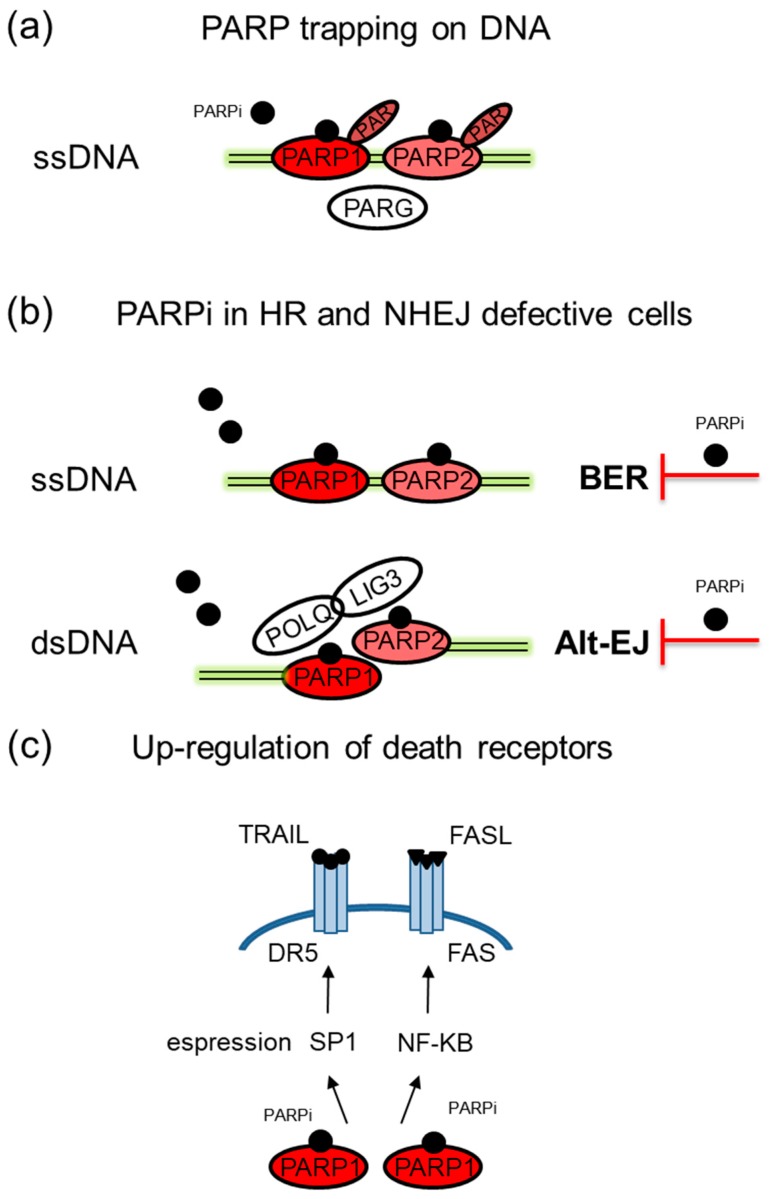
Additional mechanisms of action of PARPi. (**a**) In the presence of PARPi, PARP1/2 trapping on damaged DNA may occur with consequent formation of PARP-DNA complexes that prevent DNA replication and transcription. PARPi vary significantly in their trapping abilities. (**b**) In case of cells defective in HR and NHEJ, PARPi prevent DSB repair by Alt-EJ with induction of cell death. (**c**) PARPi favor up-regulation of death receptors through activation of SP1 and NF-kB transcription factors.

In the presence of BRCA1 mutations that impair heterodimerization with BARD1, treatment with PARPi would decrease PAR formation and the fast recruitment of the BRCA1/BARD1 complex to DNA damage sites with consequent inhibition of HR-mediated repair and induction of cell death [30]. An additional mechanism of PARPi relies on activation of NHEJ that derives from the reduced PARP1 ability to prevent KU proteins interaction with free DNA ends [45]. Aberrant activation of NHEJ may result in genomic instability and eventually cancer cell death. In case of functional defects of both HR and classical NHEJ, inhibition of PARP1 avoids activation of Alt-EJ and cancer cells undergo apoptosis [46]. Finally, up-regulation of DR5 and FAS death receptors, which requires activation of SP1 or NF-kB transcription factors, contributes to PARPi anticancer effects through tumor cell sensitization to TRAIL or FASL death receptor ligands [47,48] (Figure 2).

Initial observations in a phase I clinical study established the feasibility of the synthetic lethality approach demonstrating that the PARPi olaparib (an inhibitor of PARP1 and PARP2; Lynparza, AstraZeneca, Cambridge, UK) induced an objective antitumor activity in BRCA carriers with ovarian, breast or prostate cancers with fewer adverse effects as compared to conventional chemotherapy [49]. Results of subsequent trials confirmed the higher efficacy of PARPi use as monotherapy in BRCA mutated OC with respect to BRCA wild-type tumors [50,51,52]. In 2014, olaparib (capsule) was approved by EMA as maintenance treatment of patients with recurrent high grade serous epithelial ovarian, fallopian tube or primary peritoneal cancer with mutations in BRCA genes who are in a complete or partial response to platinum-based chemotherapy [52]. Few months later, FDA granted approval to olaparib for the treatment of germline BRCA-mutated advanced OC in patients who had previously received three or more lines of chemotherapy [53]. Recently, a tablet formulation of olaparib (that allows to reduce the daily pill burden) received approval by both EMA and FDA for the maintenance therapy of platinum-sensitive recurrent OC regardless of BRCA mutational status [52,54] (Table 1).

After the initial approval of olaparib, two other PARPi received market authorization: rucaparib (PARP1, PARP2 and PARP3 inhibitor; Rubraca, Clovis Oncology, Boulder, CO, USA) and niraparib (PARP1 and PARP2 inhibitor; Zejula, Tesaro, Waltham, MA, USA) for the treatment of BRCA-mutated OC relapsed after two or more lines of chemotherapy and/or for the maintenance therapy of platinum-sensitive, recurrent high-grade epithelial OC [55,56,57,58]. In the registration clinical trials OC, fallopian tube cancer, or primary peritoneal cancer with predominantly high-grade serous (niraparib) or high-grade serous or endometrioid (rucaparib) histological features were enrolled. According to the recently approved indications of PARPi as maintenance therapy, tumor responsiveness to cisplatin (as a surrogate marker of HR-deficiency) has substantially replaced the requirement of BRCA mutational status analysis in the tumor samples for determining patients’ eligibility.

Finally, in 2018, olaparib as well as talazoparib (highly potent PARP1 and PARP2 inhibitor clinically active at 1 mg dose; Talzenna, Pfizer, New York, NY, USA) were FDA approved for patients with epidermal growth factor receptor 2 (HER2)-negative metastatic BC with BRCA mutations, relapsing after previous chemotherapy in the neoadjuvant, adjuvant, or metastatic setting [59,60] (Table 1). In the next paragraphs, we will consider the scientific evidence that emerged from both experimental and clinical studies leading to the extended market authorization of the PARPi.

## 5. Germline and Somatic BRCA Mutation Carriers Equally Respond to PARPi

As initially reported by Gelmon and collaborators in a phase II study with olaparib, objective responses were confirmed in 41% (7 out of 17) of OC patients with germline BRCA mutations and in 24% (11 out of 46) of patients without germline mutations [51]. Remarkably, in responders belonging to the latter group BRCA somatic mutations were detected. Subsequent studies with olaparib (Study 19) [52] and with rucaparib (Ariel 2 and Study 10) [58,61] confirmed that BRCA mutated patients derive the greatest clinical benefit from PARPi treatment and showed no differences in responsiveness to PARPi between germline and somatic BRCA-mutated OC.

This has allowed increasing the number of patients who may likely benefit from the treatment with PARPi. In high-grade serous OC (HG-SOC), which is the most common histological type of epithelial OC, deleterious mutations in BRCA are detected in 17–25% of patients, somatic mutations representing 18–30% of all BRCA mutations [62,63]. Recently, Dougherty et al. analyzed by Next-Generation Sequencing (NGS) 265 patients with platinum sensitive HG-SOC (Study 19, a phase II trial with olaparib) and found BRCA deleterious mutations in 55% of tumors, 10% of which were somatic mutations (i.e., 18% of total patients). The high mutation prevalence reported in the latter study might reflect platinum responsive patient selection and the use of a more efficient NGS-based method. Of interest, 100% of germline and 83% of somatic loss-of-function mutations had biallelic inactivation and were predominantly clonal, suggesting that BRCA loss occurs early in the development of HG-SOC [64].

The rate of somatic BRCA mutations in other cancers appears to be lower. In invasive BC Winter et al. reported deleterious germline BRCA mutations in 9% of patients and somatic BRCA mutations in 3% of cases [65]. In advanced prostate cancer, recently reported BRCA mutation rates, assessed by whole genome sequencing, were 0.6% for BRCA1 and 12% for BRCA2; in all cases biallelic inactivation was present [66]. Clinical studies with PARPi are ongoing also in metastatic breast and prostate cancer with somatic deleterious BRCA mutation.

## 6. Deleterious BRCA Mutations and Sensitivity to Platinum-Based Drugs or PARPi

In vitro and in vivo preclinical studies have shown that over-expression of BRCA is associated with resistance to cisplatin [67]; on the contrary, BRCA deleterious mutations sensitize cancer cells to the platinum compound [68]. These findings were validated by clinical studies showing that BRCA mutation carriers had longer disease-free intervals and survival after platinum treatment compared with wild-type patients [69,70,71,72]. Moreover, Pennington and collaborators compared platinum sensitivity and overall survival in ovarian carcinoma patients with loss-of-function mutations in 30 genes of the HR pathway (including germline and somatic BRCA mutations) and found that mutations were highly predictive of platinum response [63]. Therefore, BRCA mutations and HR deficiency are regarded as predictive markers of high sensitivity to platinum compounds.

Since platinum responsiveness correlates with HR deficiency, it was expected that platinum-sensitive tumors would have responded to PARPi as well. Indeed, the first clinical studies with PARPi confirmed this hypothesis. In particular, in a study on germline BRCA-mutated OC, there was a significant association between tumor response to olaparib and platinum-free interval. In particular, in 46 evaluable patients, the overall clinical benefit rate was significantly higher in the platinum-sensitive group (69.2%) than in the platinum-resistant (45.8%) or platinum-refractory ones (23.1%). Platinum-resistant and platinum-refractory diseases are, respectively, defined as disease progression within 6 months of prior platinum therapy or during platinum therapy, respectively, and both conditions are associated with a poor prognosis [49]. Interestingly, Audeh and colleagues observed an objective response in 38% of platinum-sensitive patients and 30% in platinum-resistant patients treated with olaparib [50]. Although, the clinical study was not designed to compare differences in the response to platinum-based therapy, the results indicated that even platinum-resistant tumors may respond to PARPi although to a lesser extent as compared to the platinum-sensitive ones. These data were further confirmed by the results of Study 42 with olaparib that included 193 patients with germline BRCA-mutated platinum-resistant OC, not suitable for further platinum therapy. The observed tumor response rate and stable disease were 31% and 40%, respectively [53]. Thus, there is not always a cross-resistance between platinum compounds and PARPi.

## 7. Sensitivity to PARP Inhibitors beyond BRCA Mutations

Subsequent studies, in particular with the PARPi olaparib, were mostly performed in platinum responders. Ledermann et al. reported in 2014 the results of a phase II clinical trial (study 19) that enrolled 265 patients with platinum-sensitive recurrent OC to receive olaparib or placebo. The primary endpoint of the study was progression-free survival (PFS) analysis by BRCA status. Patients with BRCA mutations treated with olaparib showed a longer PFS than the placebo group (11.2 vs. 4.3 months; hazard ratio 0.18). Notably, a significant benefit in favor of olaparib was also reported in patients with wild-type BRCA (7.4 vs. 5.5 months; hazard ratio 0.54) [52]. In fact, other BRCA-dependent as well as BRCA-unrelated mechanisms might lead to HR impairment and may account for the clinical benefit obtained by patients with wild-type BRCA.

**Figure 3:**
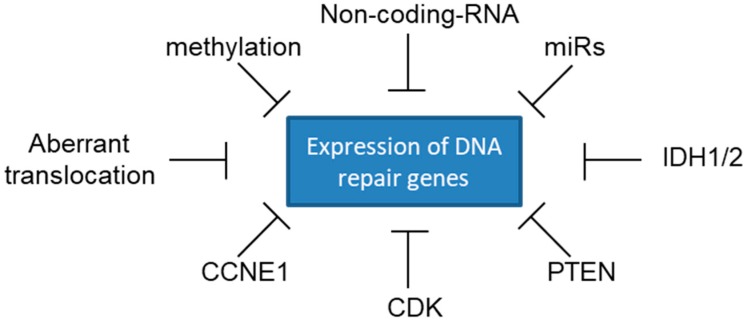
Molecular mechanisms that may cause reduced expression of DNA repair genes.

Methylated BRCA1 or BRCA2 genes [73,74], over-expression of miR-182 [75], high expression of the long noncoding RNA PCAT1 [76] are all mechanisms able to hamper mRNA translation of BRCAs and confer sensitivity to PARPi (Figure 3). In acute myeloid leukemia (AML) BRCA genes are rarely mutated [77]; nevertheless, their expression is reduced as compared to normal bone marrow [78,79]. This would account for the sensitivity to olaparib of primary AML cultures that we reported in recent studies [48,79].

Altered expression of other proteins involved in the HR pathway or in DSB sensing may also cause a “BRCAness” phenotype. Defective ATM protein or aberrations of the MRE11A-RAD50-NBN complex, essential in DNA damage sensing and detection [80,81,82], silencing or deletion of other proteins involved in HR, such as ATR, RAD51, RAD54, DSS1, RPA1, NBN, CHK1, CHK2, FANCD2, FANCA, FANCC, FANCM or BARD1, may result in tumor sensitivity to PARPi monotherapy [80,83,84]. Other members of the Fanconi anemia family, such as RAD51C, RAD51D, BRIP1 and PALB2 also confer sensitivity to DNA damage and DNA repair inhibition [83,85,86,87]. Of interest, most of these genes frequently show genomic alterations in OC as well as in other tumors [63].

Accumulating experimental evidence suggests a potential therapeutic role for PARPi also in tumor subgroups characterized by mutations in genes not directly involved in HR repair. Altered expression of upstream HR modulators, as PTEN [88] or IDH1 and IDH2 [89,90], may induce a BRCAness phenotype that would increase tumor sensitivity to PARPi. Cyclin-dependent kinases (CDKs) are involved in regulating the cell cycle, transcription and mRNA processing. In models of HG-SOC, a decrease in CDK12 caused a reduction of BRCA1 expression and suppression of HR repair, resulting in enhanced sensitivity to PARPi [91]. Overexpression of EMSY, a nuclear protein that binds to the BRCA2 N-terminal domain, can hamper BRCA2 function in DNA-damage repair [92]. Also, certain chromosomal translocations, common in AML, like t(8;21) (RUNX1-RUNXT1), t(15;17) (PML-RARA), and t(17;19) (TCF3-HLF), can reduce HR repair activity and sensitize tumor cells to PARPi [93,94] (Figure 3). Therefore, the sole analysis of BRCA mutational status is not sufficient to identify all patients with HR-deficient tumors eligible for treatment with PARPi.

A number of experimental evidences indicate that functional defects in HR induce DNA damage that can lead to loss or duplication of chromosome regions, also known as genomic loss of heterozygosity (LOH) [95]. The myChoice^®^ HRD test (Myriad^®^ Genetics, Salt Lake City, UT, USA) is an informatics test created on the basis of an unweighted sum of loss of heterozygosity (LOH), telomeric allelic imbalance (TAI) and large-scale state transitions (LST) in tumor samples to assess a combined HR deficiency score. Patients selected for a high HR deficiency score likely respond to drugs affecting DNA stability as platinum agents and PARPi [96]. This assay was used to identify HR deficiency in the phase III ENGOT-OV16/NOVA clinical trial with niraparib that enrolled patients with predominantly high-grade serous OC patients responsive to platinum-based treatment, who had received at least two previous chemotherapies [55]. Patients were divided in two cohorts, based on the presence (*n* = 180) or absence (*n* = 310) of a germline BRCA mutation, and randomly assigned to receive niraparib or placebo. Median PFS values were 21.0 vs. 5.5 months (hazard ratio 0.27) in the germline BRCA cohort and 9.3 vs. 3.9 months (hazard ratio 0.45) in the overall non-germline BRCA cohort. Niraparib efficacy in the non-germline BRCA cohort was further analyzed based on HR functional status, including or excluding somatic BRCA mutations. The median PFS values in patients treated with the PARPi with respect to placebo were: 20.9 vs. 11 months (hazard ratio 0.27) in case of HR deficiency plus somatic BRCA mutations, 9.3 vs. 3.7 months (hazard ratio 0.38) in case of HR deficiency and wild-type BRCA, and 6.9 vs. 3.8 months (hazard ratio 0.58) in the HR-proficient subgroup. These data confirm that besides BRCA mutations, other cellular defects may inhibit DSB repair and account for sensitivity to PARPi, and that niraparib showed some efficacy even in the absence of BRCA mutations or HR deficiency. Remarkably, in 20% of patients lacking HR deficiency a long-term (>18 months) clinical benefit was observed [55].

In the ARIEL2 clinical trial with rucaparib, platinum-sensitive, high-grade OC patients were classified into one of three predefined HR deficiency subgroups: BRCA mutant (deleterious germline or somatic) (*n* = 40), BRCA wild-type and LOH-high (≥14% LOH) (*n* = 82) or BRCA wild-type and LOH-low (*n* = 70) [58]. The analysis was performed in 192 pretreatment tumor biopsies containing more than 20% of tumor cells (with a minimum of 80% nucleated cellular content) by a targeted NGS-based assay. The NGS results were compared with those obtained by a second test on archival formalin-fixed paraffin-embedded tissues (*n* = 145). Samples with LOH segments spanning 90% of a whole chromosome arm were excluded, as these events usually arise through non-HR mechanisms (e.g., mitotic nondisjunction). After rucaparib treatment, the median PFS values were 12.8 months in the BRCA mutant subgroup, 5.7 months in the BRCA–wild-type/LOH-high subgroup, and 5.2 months in the BRCA–wild-type/LOH-low subgroup. The risk of tumor progression was reduced in the BRCA mutant (hazard ratio 0.27) and BRCA–wild-type/LOH-high (hazard ratio 0.62) subgroups as compared to the BRCA–wild-type/LOH-low subgroup. The percentage of patients who were progression-free at 12 months was higher in the BRCA mutant (50.4%) and BRCA–wild-type/LOH-high (28%) subgroups than in the BRCA–wildtype/LOH-low subgroup (9.6%) [58].

In the ARIEL2 study, the authors also sequenced a considerable number of biomarker genes known to be indicators of HR deficiency, with the aim of searching predictive markers of PARPi sensitivity. Moreover, they analyzed tumor sample for the BRCA1 or RAD51C methylation pattern, and presence of alterations in the main tumor suppression genes (TP53 and RB1) and in the P13K/RAS signaling [58]. The copy number of the same genes were also tested. Analysis of a tumor sample collected from a patient with BRCA–wild-type/LOH-low genotype who had a complete response to rucaparib showed amplification of cyclin E1 (CCNE1), which is able to suppress BRCA1 expression [97]. However, this alteration cannot be considered a reliable predictive biomarker, since other patients with a high copy number of CCNE1 showed stable disease or did not respond to the PARPi. In the same tumor sample a germline deleterious mutation in the DNA repair NBN gene was also detected; unfortunately, this was the only sample with defective NBN associated with complete response and no conclusion can be drawn [58]. Overall, no other clinically-relevant gene aberrations were identified and, so far, germline or somatic mutations in BRCA remain the best predictive factors to PARPi, although all groups obtained clinical benefit from rucaparib.

In the ARIEL3 phase III study Coleman and colleagues evaluated rucaparib as maintenance therapy in patients with recurrent OC who achieved a complete or partial response to platinum-based therapy. Patients (*n* = 564) were grouped in three cohorts: patients with BRCA mutations (deleterious germline or somatic mutations), patients with HR deficiencies (BRCA mutant or BRCA–wild-type/LOH-high), and the intention-to-treat population. Median PFS of patients with a BRCA-mutant cancer treated with rucaparib was 16.6 months (hazard ratio 0.23) vs. 5.4 months of the placebo group. In patients with a HR deficient tumor, PFS was 13.6 vs. 5.4 months (hazard ratio 0.32), while in the intention-to-treat population PFS was 10.8 vs. 5.4 months (hazard ratio 0.36). Interestingly, when the whole BRCA wild-type subgroup was considered, the PFS achieved with rucaparib was 9.7 and 6.7 months (hazard ratio 0.44 and 0.58) in patients with LOH-high and LOH-low, respectively, vs. 5.4 months of the placebo group. Overall, these data indicated that rucaparib maintenance therapy significantly improved PFS not only in patients with a BRCA mutation but also in those with BRCA wild-type OC. Furthermore, the authors stated that the benefit obtained from rucaparib administration was independent on both the measurable or bulky disease at baseline and the response to the last platinum treatment [56].

The above described clinical studies were all done in patients with high-grade OC responsive to platinum-based treatments. Although a direct comparison among the different PARPi cannot be made, due to differences in patient cohorts and study design, the magnitude of improvement in PFS recorded for patients harboring a BRCA mutation in their tumors is similar. Moreover, the treatment-related toxicities of the clinically approved PARPi are generally manageable. Surprisingly, all studies observed a significant increase in PFS also in patients with no detectable DNA repair deficiency. In the ARIEL2 and ARIEL3 clinical trials, although BRCA mutations or genomic LOH was associated with better tumor response than BRCA1 or RAD51C methylation, copy number profile or mutations in other relevant genes, the outcome with rucaparib was generally better than that obtained with placebo. Similarly, in the ENGOT-OV16/NOVA study, although in patients with HR-deficiency the median duration of PFS was significantly longer than in non HR-deficient patients, even the latter patients derived a benefit from niraparib treatment. Since durable responses can be identified also in the absence of BRCA mutations or HR deficiency, limiting PARPi treatment to carriers of BRCA mutations and LOH-high might exclude a number of patients likely to benefit from this type of treatment. Interestingly, two platinum-sensitive patients without germline or somatic BRCA mutations treated with olaparib for >5 years have been reported to experience exceptional long-term responses [98]. Nevertheless, there are no other approved biomarkers of response to PARPi.

## 8. Recent Approval of PARPi for Breast Cancer

The OlympiAD study led to the recent approval of olaparib in patients with HER2-negative metastatic BC who have previously been treated with chemotherapy. The randomized phase III study was conducted on 302 patients with tumors harboring germline BRCA mutations and lacking HER2 overexpression, irrespective of hormone-receptor status (i.e., estrogen or progesterone-receptor). Patients were randomized to receive olaparib or standard single-agent chemotherapy (capecitabine, eribulin, or vinorelbine in 21-day cycles). Median PFS was 7.0 months in the olaparib group vs. 4.2 months in the standard-therapy group (hazard ratio 0.58), with a follow-up of 14.5 months. The response rate was approximately double the rate in the standard-therapy group (59.9% vs. 28.8%) and the risk of disease progression or death was 42% lower in the olaparib group. Moreover, the study showed also fewer grade ≥3 adverse events in the olaparib monotherapy arm than in the standard-therapy group [59].

Comparable results were obtained with talazoparib in the EMBRACA clinical trial [60]. Patients with advanced BC and germline BRCA mutations (*n* = 431) were randomized to receive either the PARPi or standard chemotherapy (capecitabine, eribulin, gemcitabine, or vinorelbine in continuous 21-day cycles). Patients with HER2-positive disease were excluded. Median PFS was 8.6 months in the talazoparib group vs. 5.6 months in the standard-therapy group (hazard ratio 0.54) with a follow-up of 14.5 months. Objective response rates were 62.6% and 27.2% with talazoparib or standard therapy, respectively. Concerning treatment safety, hematological grade 3–4 adverse events were more common in the talazoparib group; however, myelotoxicity did not result in drug discontinuation and was manageable by dose modifications or delays.

The results of both clinical studies are encouraging although the improvement of PFS is only of few months; however, survival data are still immature to draw a definitive conclusion about treatment influence on overall survival. Since PARPi efficacy was not compared to that of platinum compounds, the OlympiAD and EMBRACA trials could not assess the relative benefits of PARPi and platinum-based chemotherapy in BC patients with germline BRCA mutation [59,60] Nevertheless, in the case of olaparib the authors stated that the observed response rate of 68% and the median PFS of 6.8 months were similar to those reported with first-line single-agent carboplatin in metastatic triple negative BC (TNBC, i.e., estrogen receptor-, progesterone receptor- and HER2 negative) [59]. Thus, further studies to compare PARPi with platinum agents in germline BRCA mutated BC are required. Moreover, it remains to be established whether HER2-negative BC harboring somatic mutations may also benefit from the treatment with PARPi.

To clarify whether also in the case BC, the presence of BRCA mutations or HR-deficiency in the tumor correlated with response to platinum compounds, Tutt and collaborators performed a phase III clinical trial in metastatic TNBC, which is characterized by mutational and rearrangement signatures indicative of abnormalities in DNA repair that resemble those of BRCA-mutated tumors. In TNBC, the BRCAness phenotype may derive from BRCA1 promoter methylation and/or low BRCA1 mRNA or protein expression, and in some cases from BRCA mutations. The study enrolled 376 patients, of whom half were treated with carboplatin and half with docetaxel. In the unselected population carboplatin and docetaxel showed similar efficacy, whereas in patients with germline mutated BRCA, carboplatin doubled the objective response rate obtained with docetaxel (68% vs. 33%). Such clinical benefit was not observed in patients with BRCA1 methylation, BRCA1 mRNA-low tumors or a high score in a HR deficiency assay [72]. Studies are required to compare platinum-based agents with PARPi and to assess whether platinum sensitivity correlate with tumor susceptibility to PARPi in this clinical setting.

## 9. Conclusions

Presently, three PARPi (olaparib, rucaparib and niraparib) are approved as maintenance therapy in platinum-sensitive high-grade OC regardless of BRCA mutations. Olaparib and rucaparib are also approved for the treatment of BRCA-mutated OC, independently of platinum sensitivity, and olaparib and talazoparib for the treatment of BC patients who have been treated with two or more lines of chemotherapy (Table 1).

Germline or somatic BRCA deleterious mutations result in defective DSB repair by HR and patients with tumors harboring BRCA mutations derive the greatest clinical benefit. Platinum sensitivity has been also linked to a defective HR system and considered a suitable surrogate marker for predicting patient response to PARPi. However, PARPi responsiveness has been observed also in some patients resistant to platinum compounds [53]; thus, relying only on platinum sensitivity to identify PARPi-eligible patients might exclude women who may benefit from treatment. The results of clinical trials have also indicated that even some patients with wild-type BRCA OC may respond to treatment with PARPi. Notably, Friedlander and collaborators reported 6-years’ olaparib maintenance treatment (study 19) approximately in 11% of PARPi-treated patients who showed long term benefit irrespective of BRCA mutational status [99]. Therefore, additional reliable markers need to be validated in clinical trials to select patients potentially responsive to PARPi, who cannot be identified solely on the basis of deleterious BRCA mutations or platinum responsiveness.

On the other hand, not all patients with BRCA mutations respond to PARPi suggesting the existence of primary resistance mechanisms. This suggests that the large numbers of alterations detected in the BRCA genes might not have the same functional consequences and impact on the response to PARPi.

Indeed, studies on tumor biopsies started to reveal molecular mechanisms responsible for primary and acquired resistance to PARPi. The most common acquired resistance mechanism to PARPi consists in secondary mutations restoring the BRCA1 or BRCA2 protein functionality. These mechanisms have been mainly investigated in preclinical studies and/or in patients resistant to platinum compounds [71,72]. Recently, secondary mutations that restored the open reading frame of BRCA or HR-related genes (RAD51C and RAD51D) were detected by NGS in OC cases at progression during treatment with rucaparib [100]. Moreover, in patients with BRCA somatic heterozygous disruption deriving from allelic deletion, tumor progression was associated with a recovery of BRCA activity due to copy number gain and/or upregulation of the remaining functional allele. Conversely, a patient with a single-copy loss of chromosome 17 and a somatic nonsense BRCA1 mutation in the remaining allele achieved a long-term response to olaparib (>7 years). In this case, deletion of the wild-type allele has rendered unlikely the restoration to a functional gene [98]. Consequently, integrated mutational and expression analyses may also provide an indication of the magnitude of long-term benefit from maintenance therapy with PARPi.

Additional clinically relevant resistance mechanisms may include mutations or down-regulation of PARP enzymes [101]. Indeed in a case of OC resistant to PARPi, a PARP1 mutation that prevented enzyme trapping on DNA was described [100].

HR gene sequencing, HR deficiency and LOH tests or the myChoice HRD test of Myriad Genetics scoring system allow identification of a substantial percentage of eligible patients, but they may still miss some women who might benefit from treatment. Ongoing and future clinical trials should incorporate all the information deriving from DNA sequence and copy number variation of genes involved in repair pathways other than HR (NHEJ and alt-NJ), DNA damage regulatory genes and DNA-repair related PARPs. The possibility to analyze genome-sequencing of tumor DNA by specialized high-skilled companies with sustainable costs together with advances in bioinformatics may likely give us a more complete panel of genes to define responder patients.

## Figures and Tables

**Table 1:** FDA and EMA approval of PARP inhibitors.

PARPi	Approved Indications		Registration Clinical Trial	Deleterious Germline or Somatic BRCA Mutations	Dose
	FDA	EMA			
Olaparib (Lynparza^®^, AstraZeneca, Cambridge, UK)	19 December 2014 Treatment of adult patients with deleterious or suspected deleterious germline BRCA-mutated advanced ovarian cancer who have been treated with three or more prior lines of chemotherapy.	24 October 2014 Maintenance treatment of adult patients with relapsed, platinum-sensitive high-grade serous epithelial ovarian, fallopian tube or primary peritoneal cancer with mutations (germline or somatic) in BRCA genes, and who are in response to platinum-based chemotherapy	FDA: Study 42, Phase II (NCT01078662)	Required	FDA: 400 mg PO bid (sixteen 50 mg hard capsules); in 2017: 300 mg PO bid (four 150 mg tablets)
			EMA: Study 19, Phase II (NCT00753545)		EMA: 400 mg PO bid (sixteen 50 mg hard capsules)
	17 August 2017 Maintenance treatment of adult patients with recurrent epithelial ovarian, fallopian tube or primary peritoneal cancer, who are in a complete or partial response to platinum-based chemotherapy	8 May 2018 Maintenance therapy for patients with platinum-sensitive relapsed high-grade, epithelial ovarian, fallopian tube, or primary peritoneal cancer who are in complete response or partial response to platinum-based chemotherapy, regardless of BRCA status	SOLO2, Phase III (NCT01874353) Study 19, Phase II (NCT00753545)	Not required	300 mg PO bid (four 150 mg tablets)
	12 January 2018 Treatment of patients with deleterious or suspected deleterious germline BRCA-mutated, human epidermal growth factor receptor 2 (HER2)-negative metastatic breast cancer who have previously been treated with chemotherapy in the neoadjuvant, adjuvant or metastatic setting.		OlympiAD, Phase III (NCT02000622)	Required	300 mg PO bid (four 150 mg tablets)
Rucaparib (Rubraca^®^, Clovis Oncology, Boulder, CO, USA)	19 December 2016 Treatment of adult patients with deleterious BRCA mutation (germline and/or somatic)-associated epithelial ovarian, fallopian tube, or primary peritoneal cancer who have been treated with two or more chemotherapies.	24 May 2018 Treatment of adult patients with platinum sensitive, relapsed or progressive, BRCA-mutated (germline and/or somatic), high-grade epithelial ovarian, fallopian tube, or primary peritoneal cancer, who have been treated with two or more prior lines of platinum based chemotherapy, and who are unable to tolerate further platinum based chemotherapy.	ARIEL 2, Phase II (NCT01891344); Study 10, Phase I/II (NCT01482715)	Required	600 mg PO bid (four 300 mg film/coated tablets)
	6 April 2018 Maintenance treatment of adult patients with recurrent epithelial ovarian, fallopian tube, or primary peritoneal cancer who are in a complete or partial response to platinum-based chemotherapy		ARIEL3, Phase III (NCT01968213)	Not required	
Niraparib (Zejula^®^, Tesaro, Waltham, MA, USA)	27 March 2017 Maintenance treatment of adult patients with recurrent epithelial ovarian, fallopian tube, or primary peritoneal cancer who are in a complete or partial response to platinum-based chemotherapy	16 November 2017 Maintenance treatment of adult patients with platinum-sensitive relapsed high grade serous epithelial ovarian, fallopian tube, or primary peritoneal cancer who are in response (complete or partial) to platinum-based chemotherapy	NOVA study, phase III (NCT01847274)	Not required	300 mg PO qd (three 100 mg hard capsules)
Talazoparib (Talzenna^®^, Pfizer Inc., New York, NY, USA)	16 October 2018 Treatment of adult patients with deleterious or suspected deleterious germline BRCA-mutated HER2-negative locally advanced or metastatic breast cancer.		EMBRACA, phase III (NCT01945775)	Required	1 mg PO qd (1 hard capsule)

## Abbreviations

APEX1	apurinic/apyrimidinic endodeoxyribonuclease 1
ATM	ATM serine/threonine kinase
ATR	ATR serine/threonine kinase
BARD1	BRCA1 associated RING domain 1
BRCA1	BRCA1, DNA repair associated
BRCA2	BRCA2, DNA repair associated
BRIP1	BRCA1 interacting protein C-terminal helicase 1
CCNE1	cyclin E1
CDK12	cyclin dependent kinase 12
CHK1	checkpoint kinase 1
CHK2	checkpoint kinase 2
CtIP	official name: RBBP8, RB binding protein 8, endonuclease
DSS1	SEM1, 26S proteasome complex subunit
DR5	official name: TNFRSF10B, TNF receptor superfamily member 10b
EMSY	EMSY, BRCA2 interacting transcriptional repressor
FANCA	FA complementation group A
FANCC	FA complementation group C
FANCD2	FA complementation group D2
FANCM	FA complementation group M
FAS	Fas cell surface death receptor
H2AX	official name: H2AFX, H2A histone family member X
γH2AX	phospho-H2AFX
IDH1	isocitrate dehydrogenase (NADP+) 1, cytosolic
IDH2	isocitrate dehydrogenase (NADP(+)) 2, mitochondrial
KU70	official name: XRCC6, X-ray repair cross complementing 6
KU80	official name: XRCC5, X-ray repair cross complementing 5
LIG3	DNA ligase 3
LIG4	DNA ligase 4
MRE11	MRE11 homolog, double strand break repair nuclease
NBN	nibrin (also known as NBS1)
NF-kB	official name: REL proto-oncogene, NF-kB subunit
PALB2	partner and localizer of BRCA2
PARG	poly(ADP-ribose) glycohydrolase
PARP1	poly(ADP-ribose) polymerase 1
PARP2	poly(ADP-ribose) polymerase 2
PARP3	poly(ADP-ribose) polymerase family member 3
POLB	DNA polymerase β
POLD POLL POLM	DNA polymerase δ DNA polymerase λ DNA polymerase µ
POLQ	DNA polymerase θ
PTEN	phosphatase and tensin homolog
RAD50	RAD50 double strand break repair protein
RAD51	RAD51 recombinase
RAD51C	RAD51 paralog C
RAD51D	RAD51 paralog D
RAD52	RAD52 homolog, DNA repair protein
RAD54	RAD54 homolog B
RB1	RB transcriptional corepressor 1
RIF1	replication timing regulatory factor 1
RPA1	replication protein A1
SP1	Sp1 transcription factor
TP53	tumor protein p53
TP53BP1	tumor protein p53 binding protein 1
PCAT1	prostate cancer associated transcript 1 (non-protein coding)
XLF	official name: NHEJ1, non-homologous end joining factor 1
XRCC1	X-ray repair cross-complementing 1
XRCC4	X-ray repair cross complementing 4
Alt-EJ	alternative Non-homologous End Joining (also known as alt-NHEJ, a-NHEJ, back-up NHEJ, B-NHEJ, MMEJ)
BER	base excision repair
DSB	DNA double-strand breaks
HR	homologous recombination
NGS	Next-Generation Sequencing
NHEJ	Non-homologous End Joining
SSB	single strand break
